# Modulation of photocarrier relaxation dynamics in two-dimensional semiconductors

**DOI:** 10.1038/s41377-020-00430-4

**Published:** 2020-11-23

**Authors:** Yuhan Wang, Zhonghui Nie, Fengqiu Wang

**Affiliations:** 1 School of Electronic Science and Engineering and Collaborative Innovation Center of Advanced Microstructures, Nanjing University, Nanjing, 210093 China; 2 Key Laboratory of Intelligent Optical Sensing and Manipulation, Ministry of Education, Nanjing University, Nanjing, 210093 China; 3 MIIT Key Laboratory of Advanced Display Materials and Devices, Institute of Optoelectronics & Nanomaterials, College of Materials Science and Engineering, Nanjing University of Science and Technology, 210094 Nanjing, China

**Keywords:** Optical materials and structures, Optoelectronic devices and components

## Abstract

Due to strong Coulomb interactions, two-dimensional (2D) semiconductors can support excitons with large binding energies and complex many-particle states. Their strong light-matter coupling and emerging excitonic phenomena make them potential candidates for next-generation optoelectronic and valleytronic devices. The relaxation dynamics of optically excited states are a key ingredient of excitonic physics and directly impact the quantum efficiency and operating bandwidth of most photonic devices. Here, we summarize recent efforts in probing and modulating the photocarrier relaxation dynamics in 2D semiconductors. We classify these results according to the relaxation pathways or mechanisms they are associated with. The approaches discussed include both tailoring sample properties, such as the defect distribution and band structure, and applying external stimuli such as electric fields and mechanical strain. Particular emphasis is placed on discussing how the unique features of 2D semiconductors, including enhanced Coulomb interactions, sensitivity to the surrounding environment, flexible van der Waals (vdW) heterostructure construction, and non-degenerate valley/spin index of 2D transition metal dichalcogenides (TMDs), manifest themselves during photocarrier relaxation and how they can be manipulated. The extensive physical mechanisms that can be used to modulate photocarrier relaxation dynamics are instrumental for understanding and utilizing excitonic states in 2D semiconductors.

## Semiconductor photocarriers: Exploring excitons in two dimensions

Electrons and positively charged regions in semiconductors known as holes can form tightly bound states called excitons, offering new opportunities in optoelectronics and in ‘valleytronics,’ which exploits the effects of valleys and peaks in an energy landscape. The excitons display particle-like properties, so are described as quasiparticles. Fengqiu Wang and colleagues at Nanjing University, in China, review many technical aspects of the creation and control of excitons in single layer (2D) semiconductor sheets. The restrictions of 2D materials promote unique exciton behavior and strong light-matter interactions. The authors focus on the ability of excitons to act as photocarriers of absorbed light energy, and on the relaxation processes that can release the energy. Learning how to control exciton formation, interactions and relaxation will be crucial for using them to develop novel optoelectronic and photonic devices.

## Introduction

Due to the reduced dielectric screening and enhanced quantum confinement, the Coulomb interaction experienced by carriers in 2D semiconductors is substantially increased compared to three-dimensional (3D) semiconductors, leading to the formation of tightly bound excitons and strong many-body effects^1,2^. As a result, 2D semiconductors can host a rich set of excitonic species, which exhibit large oscillator strengths and strong light-matter interactions^3,4^. Moreover, the flexibility in constructing van der Waals (vdW) heterostructures further enriches the excitonic physics in 2D semiconductors by enabling various interlayer species and introducing exotic effects such as moiré potential patterns^5^. In addition, the broken inversion symmetry of 2D transition metal dichalcogenides (TMDs) renders the electronic “valleys” non-degenerate, giving the excitons another “valley” degree of freedom with direct optical accessibility^6,7^.

Typically, excitons are generated by photoexcitation and dominate the optical properties of 2D semiconductors. Thus, probing the dynamics of the optically generated excited states represents an important aspect of excitonic physics in 2D semiconductors. Moreover, because of the low dimensionality, the excitonic dynamics of 2D semiconductors can be more susceptible to various external stimuli, opening up ways for flexible excited-state lifetime control. This is beneficial for practical applications, as excited-state lifetimes are linked to key figures of merit of multiple optoelectronic and photonic devices. For example, while a short photocarrier lifetime is favorable for the operating bandwidth of ultrafast optical switches^8^, an increase in the radiative lifetime of photocarriers has been found to coincide with the enhancement of the luminescence quantum yield (QY), which is beneficial for light-emitting devices^9^. In the case of photoconductors, the photocarrier lifetime can be a knob to balance the response time and quantum efficiency: shorter lifetimes of photocarriers can lead to a faster response speed at the expense of quantum efficiency, as more photocarriers recombine before entering the circuit^10^. Hence, exploring deterministic tuning strategies for the dynamic characteristics of photocarriers in 2D semiconductors is of both fundamental relevance and practical significance.

To identify robust and deterministic approaches for photocarrier lifetime control, experiences from conventional bulk semiconductor research can be considered, including element doping^11,12^, composition control^13^, morphology control of nanostructures^14,15^, external field application^16^, etc. Taking GaAs, one of the most widely applied conventional semiconductors, for example, modulation of its photocarrier lifetime has been achieved over a large range, from nanoseconds to sub-picoseconds, by means of low-temperature growth^17,18^ or ion implantation^19,20^. Combined with post-growth thermal annealing, these methods can effectively tune the photocarrier lifetimes without obviously degrading the crystallinity, making GaAs highly competitive in the application of ultrafast optoelectronic and photonic devices. However, due to the atomic thickness feature, in many cases, the approaches for bulk samples are difficult to adapt to or become ineffective for 2D semiconductors. For instance, the difficulty in introducing point defects or dopants into the lattice in a controllable manner is significantly enhanced. On the other hand, the unique properties of 2D semiconductors, such as robust excitonic states, sensitivity to external environmental factors, and flexibility in constructing vdW heterostructures, promise modulation strategies different from those for conventional materials.

Since there have been a number of review articles on the ultrafast photocarrier dynamics in 2D semiconductors^21–23^, in this review, we focus on summarizing recent efforts in identifying methods to modulate the photocarrier relaxation behavior. We start with a brief introduction to the photocarrier relaxation dynamics in 2D semiconductors and then devote a section to discussing modulation of Coulomb interactions and the resulting effects on the transient properties of 2D semiconductors. Subsequently, we discuss the factors that can influence photocarrier relaxation in 2D semiconductors and corresponding modulation methods, according to the related relaxation pathways or mechanisms. The tailoring approaches discussed include both those arising from the experience with bulk semiconductors, such as doping and applying external fields, and those utilizing the unique properties of 2D materials, such as modulating the surrounding environment and constructing heterostructures. After that, modulation of the spin/valley polarization dynamics is discussed as an important complement to the case of 2D TMDs. Finally, we provide a summary and an outlook on the research directions in the future.

## Photocarrier relaxation dynamics in 2D semiconductors

**Fig. 1:**
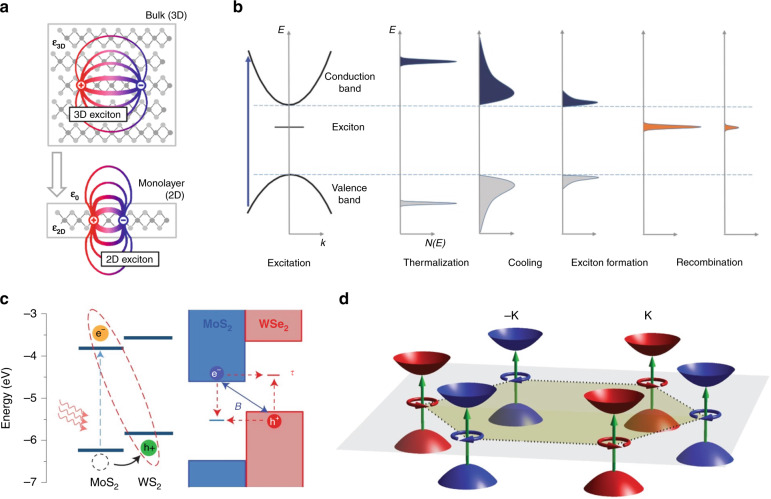
Photocarrier relaxation dynamics in 2D semiconductors. **a** Real-space representation of electrons and holes bound into excitons for a 3D bulk and a quasi-2D monolayer. **b** Evolution of photocarriers after instantaneous interband excitation. **c** (Left) Schematic of the type-II band alignment of a MoS_2_/WS_2_ heterostructure and the charge transfer process. (Right) Band profile of a MoS_2_/WSe_2_ heterostructure illustrating the mechanisms of interfacial recombination: the Shockley–Read–Hall (SRH) mechanism (red dashed arrow) and the Langevin mechanism (blue arrow). **d** Valley-contrasting optical selection rules in a 2D hexagonal lattice with broken inversion symmetry. Figure reproduced with permission from **a** ref. ^24^, © 2014 American Physical Society; **b** ref. ^22^, © 2016 WILEY‐VCH Verlag GmbH & Co., KGaA, Weinheim; **c** ref. ^29^, © 2014 Springer Nature and ref. ^31^, © 2014 Springer Nature; **d** ref. ^34^, © 2014 Springer Nature

As the sample thickness is reduced from the bulk to the atomic level, electrons and holes are tightly bound together, forming excitons, due to the greatly enhanced Coulomb interactions compared to the 3D counterparts (Fig. 1a). The resulting binding energies can be as large as hundreds of millielectronvolts for 2D semiconductors with large effective carrier masses such as TMDs^1,24^. The optical and optoelectronic properties of 2D semiconductors are dominated by the excitonic states even at room temperature.

The photocarrier dynamics in 2D semiconductors have been actively investigated in recent years, which has been covered in a number of reviews^21–23^. Once optically injected into the samples, the non-equilibrium photocarriers typically undergo relaxation processes, including rapid thermalization through carrier-carrier scattering, cooling to the band edges through interaction with phonons, and electron–hole recombination either directly or via the assistance of defects or phonons (Fig. 1b)^22^. It is worth noting that the photocarriers that relax to the band edges tend to form excitons within ~1 ps before they recombine, which has been demonstrated both experimentally and theoretically^25,26^. Meanwhile, excitons can also be injected directly by optical excitation at the excitonic resonance^21,27^.

In a vdW heterostructure formed by 2D materials, generally, after interband photoexcitation, the photocarriers in the vdW heterostructure relax the following two main steps, as illustrated in Fig. 1c. Within the first picosecond, the excited carriers transfer across the vdW interface and relax to the lowest available energy states in the band structure, i.e., the conduction band minimum for electrons and valence band maximum for holes^28^. In a MoS_2_/WS_2_ heterostructure, interfacial charge transfer has been found to occur within ~50 fs^29^. Moreover, by studying MoS_2_/WSe_2_ heterostructures with different angular alignments, Zhu et al.^30^ revealed that this charge transfer process is independent of the momentum mismatch between the two layers owing to the excess energy of the photocarriers. Due to the strong Coulomb coupling, the separated electrons and holes can still form excitons across the vdW interface, called interlayer excitons. On longer timescales, the relaxation dynamics are dominated by the recombination of interlayer excitons^28^. Interfacial recombination can occur via two possible mechanisms or their combination: Shockley–Read–Hall (SRH) recombination and Langevin recombination^31^. SRH recombination is a monomolecular process mediated by tunneling of majority carriers to trap states, and Langevin recombination is a bimolecular process dominated by the Coulomb interaction, whose rate increases with the carrier mobility^32,33^.

In addition, as a unique property of monolayer TMDs, charge carriers can carry another degree of freedom—valley polarization. Because of the broken inversion symmetry of monolayer TMDs, the valleys of energy-momentum dispersion at the corner of the hexagonal Brillouin zone, labeled +K and −K valleys, are no longer equivalent (Fig. 1d). The interband transitions at +K/−K valleys are coupled to photons with $\sigma ^ + /\sigma ^ -$ (right/left) circular helicity, allowing for optical creation, manipulation, and detection of the valley index^34^. Moreover, due to the strong spin-orbit coupling of charge carriers at the band-edge, the spin index is locked with the valley index and thus can be accessed by helicity-resolved optical approaches. For recent advances in valley-contrasting physics and applications, readers are referred to refs. ^34–38^.

## Coulomb interactions in 2D semiconductors

The enhanced Coulomb interactions in 2D semiconductors can be modulated by introducing additional screening from the external dielectric environment or injected charge carriers, leading to a modification of quasiparticle bandgaps (bandgap renormalization) and a decrease in the exciton binding energy^39,40^. These two effects partially cancel each other out and give rise to a comparatively small shift of the excitonic resonance, and the actual shift of the resulting optical bandgap may vary for different materials and conditions^41^.

### Screening induced by the dielectric environment

**Fig. 2:**
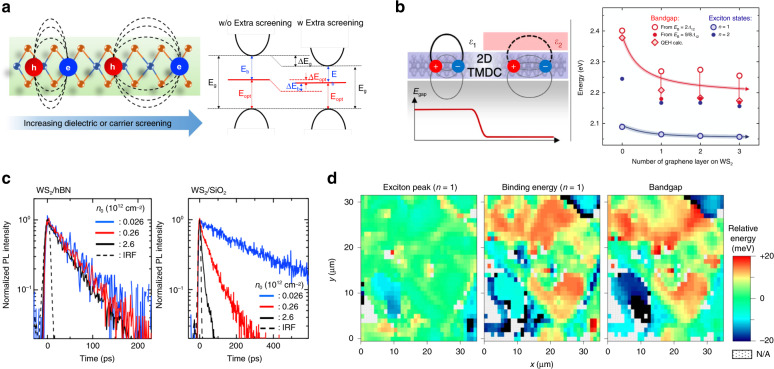
Dielectric screening of Coulomb interactions. **a** (Left) Illustration of increased screening of Coulomb interactions in 2D semiconductors. (Right) Schematic illustration showing the impact of increased screening of Coulomb interactions on the electronic bandgap (*E*_g_), exciton binding energy (*E*_b_), and optical bandgap (*E*_opt_) of 2D semiconductors. **b** (Left) Schematic illustration of a semiconducting 2D TMD material partially covered with an ultrathin dielectric layer. (Right) Absolute energies of experimentally measured excitonic states and estimated positions of the bandgap as functions of the number of layers of capping graphene. **c** Normalized exciton PL decay signals for (left) WS_2_/*h*BN and (right) WS_2_/SiO_2_ at different initial exciton densities. **d** (From left to right) Fluctuations of the *n* = 1 exciton peak position, fluctuations of the *n* = 1 exciton binding energy, and correlated fluctuations of the free-particle bandgap of an *h*BN-encapsulated WS_2_ sample exhibiting spatial variations. Figure reproduced with permission from **b** ref. ^49^, © 2017 Springer Nature; **c** ref. ^43^, © 2017 American Physical Society; **d** ref. ^51^, © 2019 Springer Nature

Research efforts have been made to alter the dielectric environment of 2D semiconductors by encapsulating 2D semiconductors with boron nitride (*h*BN)^42,43^ or using solvents^44,45^ or substrates with different dielectric constants^46–48^. Fig. 2b illustrates a strategy to modulate the local dielectric environment of 2D materials. By capping 2D samples (monolayer WS_2_ and WSe_2_) using graphene and *h*BN with different thicknesses, tuning of the electronic bandgap and exciton binding energy by as much as several hundred millielectronvolts has been achieved^49^. The energy modification realized by this strategy is large enough to drive the directional in-plane motion of excitons. In the study by Hao et al., a lateral heterostructure was formed by covering part of monolayer MoSe_2_ with *h*BN. The energy offset caused by the difference in the local dielectric screening was large enough to drive the transport of excitons across the lateral junction, resulting in an initial transport speed of ~10 nm/ps^50^.

Modification of different photocarrier relaxation processes induced by such approaches has also been observed, including exciton formation^42^, exciton–exciton annihilation (EEA)^43,46,47^, and recombination^42,47^. It has been reported that as the substrate dielectric constant increases from 1.45 (quartz) to 2.5 (SrTiO_3_), the EEA rate of MoS_2_ decreases by 2 orders of magnitude, from 0.8 cm^2^/s for quartz to 0.005 cm^2^/s for SrTiO_3_. This modulation has been attributed to a change in the energy distribution of defect states induced by the screening from substrates with different permittivities, affecting the interaction between the defect-trapped excitons and mobile excitons in a defect-assisted Auger-like process^46^. Similarly, *h*BN encapsulation has also been reported to suppress the EEA process, where the EEA rate of WS_2_ has been reduced by ~20 times (Fig. 2c)^43^. However, approaches to alter the dielectric environment also induce other effects at the same time, such as modification of interfacial defect states^42^ and phononic environments^47^, and close examination is needed to determine the dominant mechanism underlying the induced modulation of photocarrier dynamics.

Moreover, it has recently been discovered that the random fluctuation of the external dielectric environment can act as a source of disorder, leading to the spatial variation of the modification of the local bandgap and exciton binding energy by tens of millielectronvolts (Fig. 2d). The dielectric homogeneity can be improved by *h*BN encapsulation, as confirmed by the observed narrowing of the linewidth of excitonic states and efficient exciton transport^51^. These findings further strengthen the impact of Coulomb interactions on the optical and transport properties of excitons and the necessity to obtain better control of them.

### Screening induced by charge carriers

Coulomb interactions can be appreciably screened by the presence of high-density charge carriers. Modification of the quasiparticle electronic bandgap and exciton binding energy can be observed when the carrier density reaches ~10^12^/cm^2^ through electrostatic doping or optical excitation^40,52,53^. Recently, it was measured by scanning tunneling spectroscopy that continuous, wide range (~200 meV) tuning of the electronic bandgap and exciton binding energy could be achieved in a ReSe_2_ monolayer placed on a back-gated graphene device, which was attributed to the tuning of Coulomb interactions by gate-controlled free carriers in graphene^54^. A decreased exciton binding energy could result in a reduction of the exciton oscillator strength. This effect has been observed in a WS_2_ field-effect transistor (FET) embedded in a microcavity, where 6–7 times tuning of the exciton oscillator strength was realized by varying the electrostatic doping level, manifesting as changes in PL and reflection intensities^55^.

**Fig. 3:**
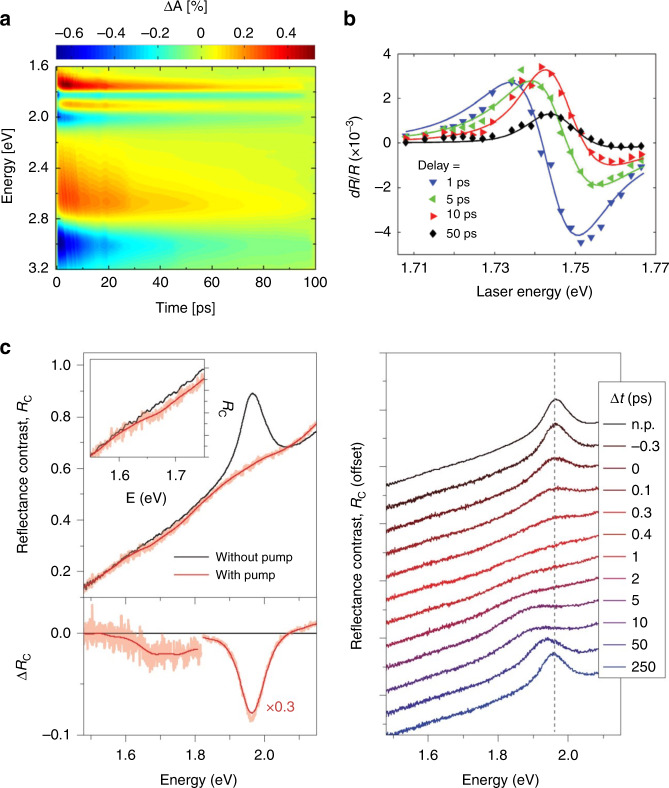
Screening of Coulomb interactions by charge carriers. **a** Transient absorption map of monolayer MoS_2_ as a function of probe photon energy and pump-probe delay after photoexcitation at 3.10 eV. **b** d*R*/*R* spectra of a WSe_2_ monolayer. **c** Photoinduced optical response of WS_2_ bilayers. (Left top) Reflectance contrast spectra without pump excitation (*R*_C,0_) and 0.4 ps after excitation (*R*_C_) by a pump pulse with an applied fluence of 840 μJ/cm^2^. (Left bottom) Corresponding differential reflectance contrast spectra Δ*R*_C_ = *R*_C_ – *R*_C,0_. (Right) Reflectance contrast spectra at different time delays after excitation. Figure reproduced with permission from **a** ref. ^56^, © 2016 American Chemical Society; **b** ref. ^57^, © 2014 IOP Publishing; **c** ref. ^39^, © 2015, Springer Nature

Under such a density of injected charge carriers, the transient optical responses of 2D semiconductors would also be modified. In the study by Cunningham et al., it was discovered that the bandgap renormalization induced by photocarriers at an excitation intensity of (2–3) × 10^12^/cm^2^ could lead to an appreciable reduction of the electronic bandgap and exciton binding energy, manifesting as bleaching of excitonic features and redshifted absorption sidebands at all excitonic resonances regardless of the excitation photon energy (Fig. 3a)^56^. In a similar study on WS_2_, the exciton binding energy was tuned from 320 to 220 meV as the absorbed fluence varied from 3 × 10^11^ to 1.2 × 10^12^ cm^2^ ^27^. Moreover, in a later study on monolayer WS_2_, the modification of the transient absorption spectra induced by high-density excitation (∼10^13^ photons/cm^2^ per pulse) included a transition from an asymmetric to symmetric profile and a blueshift of the zero differential reflectance position (Fig. 3b). It has been proposed that while exciton–exciton interaction leads to a blueshift of the excitonic resonance, a redshift can be induced by the free carriers due to the bandgap renormalization. As the lifetime of excitons (several ps) is much shorter than that of free carriers (several tens of ps), the transient optical response is dominated by free carriers on the longer timescale^57^.

As the density of injected carriers further increases to 10^13^–10^14^/cm^2^, 2D semiconductors undergo a so-called Mott transition from the semiconducting to the metallic state. A threshold carrier density of several 10^13^ cm^2^ for complete ionization (Mott transition) was estimated by extrapolating the variation in the exciton binding energy determined from reflectance contrast spectra in a study on electrostatic doping of WS_2_^40^. The signatures of the Mott transition in transient optical responses have been reported in monolayer TMDs^39^ and in TMD heterostructures where electrons and holes are confined in different TMD layers after the transition^58^. The features of excitonic resonance disappear, and a broad range of absorption emerges below the renormalized bandgap along with an optical gain just above the bandgap due to the presence of an electron–hole plasma and induced population inversion (Fig. 3c)^39,58^. These modifications are all reversible. As the photocarrier population decays with time, the excitonic feature starts to be restored after a few picoseconds, and the initial optical response can be fully recovered in hundreds of picoseconds^39^. In a subsequent study on the MoSe_2_/WSe_2_ heterostructure, the relaxation and diffusion processes of the photogenerated high-density interlayer excitons/plasma were investigated by time and spatially resolved PL. As revealed by the extracted diffusivity of the photocarriers, the electrons and holes of the plasma condense to form interlayer excitons and eventually become localized by the moiré potential as the density of photocarriers decreases during the relaxation^59^.

## Modulation of different photocarrier relaxation pathways

### Initial distribution of photocarriers in electronic band structures

When photocarriers are generated in 2D semiconductors, their initial states of occupancy in the electronic conduction band can strongly affect their decay processes by enabling available relaxation pathways in the energy and momentum space.

For example, in an ultrafast study on the transient intraband response of non-resonantly photoexcited WSe_2_, it was found that while the majority of injected free photocarriers form excitons on a sub-picosecond timescale, ~30% of them are still in the electron–hole plasma condition after several picoseconds^60^. These long-lived non-equilibrium electron–hole systems require further investigation, not only of the relaxation dynamics pertinent to free carriers but also of their influence on the relaxation of other excitonic states.

**Fig. 4:**
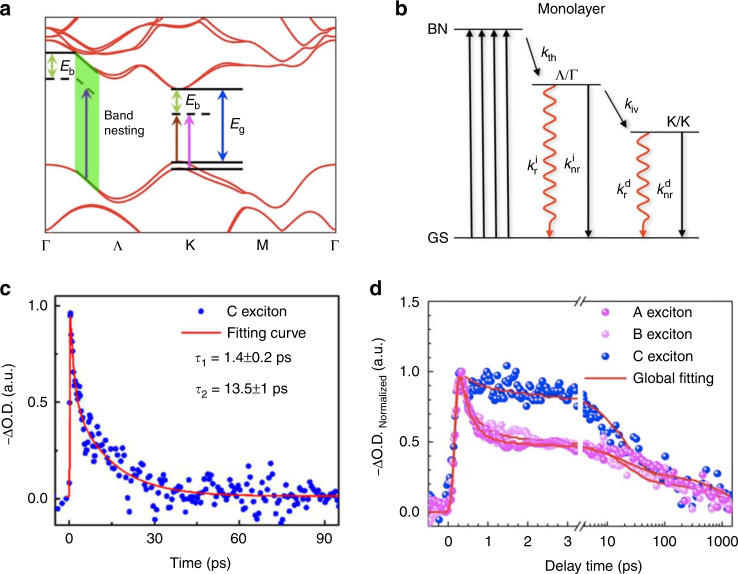
Effects of the initial distribution of photocarriers in the electronic band structure. **a** Electronic band structure of monolayer TMDs obtained by DFT calculation. The green area shows the band nesting region. **b** Relaxation pathways of photocarriers in monolayer TMDs, where the excitation is from the ground state (GS) to the band nesting (BN) region. **c** Decay dynamics of the C exciton states of monolayer MoS_2_ on a sapphire substrate after 400 nm excitation (pump density: 1.3 µJ/cm^2^, initial exciton density: 0.33 × 10^12^/cm^2^). **d** Normalized decay dynamics of the A, B, and C exciton states of monolayer MoS_2_ on a SiO_2_/Si substrate after 400 nm excitation (pump density: 5 µJ/cm^2^, initial exciton density: 1.28 × 10^12^/cm^2^). Figure reproduced with permission from **a**, **d** ref. ^61^, © 2017, Springer Nature; **b** ref. ^62^, © 2014, Springer Nature**; c** ref. ^63^, © 2019 WILEY‐VCH Verlag GmbH & Co., KGaA, Weinheim

Another example is the relaxation dynamics of higher lying states, such as the C excitons in 2D TMDs. For monolayer TMDs, the strong light absorptance has contributions from the band nesting region at the parallel bands midway between the Λ and ${\mathrm{{\Gamma}}}$ points, i.e., C excitons (Fig. 4a)^61^. It is expected that the parallel band structure in this region would promote simultaneous separation of electrons and holes and their ultrafast relaxation to immediate band extrema (Λ valley and ${\mathrm{{\Gamma}}}$ hills) with opposite momentum in *k*-space, thus suppressing the direct radiative recombination (illustrated in Fig. 4b)^62^. Such relaxation pathways have also been employed to interpret the biexponential decay dynamics of C excitons showing a fast component on the order of several picoseconds and a slow component on the order of tens of picoseconds in a study where monolayer MoS_2_ was excited by a non-resonant 400 nm pump pulse (Fig. 4c). It is proposed that the relaxation of C excitons is limited by the intervalley scattering of carriers from the Λ valley/${\mathrm{{\Gamma}}}$ hills to K valley/hills, and the observed two relaxation components represent two phonon-assisted scattering processes with different rates^63^.

In addition, it has been found that the relaxation dynamics of C excitons are also affected by the complex Coulomb environment. In the study by Wang et al., C excitons demonstrated slower relaxation processes than band-edge excitons, especially during the first tens of picoseconds, as shown in Fig. 4d. The unexpected ultralong relaxation component of C excitons was attributed to the transient and complex excited-state Coulomb environment induced by the occupied band-edge states. To prove this, the sample was excited at band-edge states and probed at the C exciton resonance, where C excitons were still observed due to an efficient up-conversion process. Under both 400 nm and band-edge excitation, the relaxation of C excitons demonstrated similar dynamics with an average lifetime of ~350 ps^61^. The longer lifetime of C excitons in this study compared with that in the study of Fig. 4c might be due to the lower density of defects in the sample. With a high density of defects, the band-edge excitons can be captured on a sub-picosecond timescale, making it difficult to induce the excited-state Coulomb environment. While the mechanisms behind the slow cooling of C excitons may need to be further elucidated, the slowing down of the cooling process is beneficial to the extraction of hot carriers. An extraction efficiency as high as 80% has been achieved in a monolayer MoS_2_/graphene heterostructure for C excitons^61^.

### Defect-assisted relaxation

**Fig. 5:**
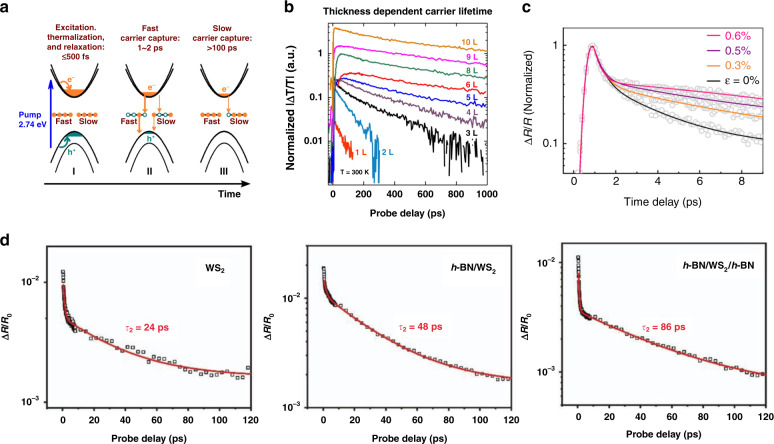
Defect-assisted photocarrier relaxation. **a** Illustration of the ultrafast carrier dynamics in MoS_2_ mediated by defect-assisted Auger recombination with two different deep mid-gap defect levels. **b** Differential transmission transients (Δ*T*/*T*) of MoS_2_ with different numbers of layers. **c** Strain-dependent transient absorption spectra of a MoS_2_ monolayer. **d** Differential reflection signal measured from WS_2_, *h*BN/WS_2_, and *h*BN/WS_2_/*h*BN regions over long-time ranges. Figure reproduced with permission from **a** ref. ^65^, © 2015, American Chemical Society; **b** ref. ^66^, © 2015 American Chemical Society; **c** ref. ^75^, © 2020 Chinese Physical Society and IOP Publishing Ltd; **d** ref. ^42^, © 2019 WILEY‐VCH Verlag GmbH & Co. KGaA, Weinheim

Scattering by defects represents an important pathway through which the non-equilibrium photocarriers lose their excess energy. In semiconductors, the mid-gap defect states can act as recombination centers or carrier traps during photocarrier relaxation, depending on the difference between the capture rates of electrons and holes by the defects^64^. Wang et al. proposed a defect-assisted recombination model describing photocarrier relaxation in monolayer MoS_2_, as illustrated in Fig. 5a. According to this model, the electrons and holes are captured by the defect states via Auger-type processes, and the two relaxation processes with timescales of ~2 and ~100 ps have been attributed to defect states located at different depths within the bandgap^65^. Furthermore, investigation of MoS_2_ with different numbers of layers revealed an increase in the photocarrier lifetime from ~50 ps to ~1 ns as the layer number increased from 1 to 10 (Fig. 5b). Such a modulation effect has been ascribed to the difference in the defect densities of surface and inner layers in that layers with higher defect densities would have a faster defect-assisted recombination rate^66^. For the case in which lattice defects act by trapping carriers, the transient reflection spectra are typically characterized by a fast switch from negative to positive^67^. Further investigation showed that when oxygen atoms acquire metal vacancies upon air exposure, the resulting defects belong to this type^68^.

Considerable efforts have been devoted to defect engineering in 2D semiconductors. Irradiation by different particle sources, such as electrons^69^, Ar^+^ plasma^68^, and Ga ions^70^, has been proven effective in creating vacancies in 2D TMD lattices, and it has been found that such irradiation favors chalcogen vacancy production due to the larger cross-section (lower atomic mass) of selenium and sulfur compared to transition metals^71^. By using electron-beam irradiation, Moody et al.^69^ demonstrated that Se vacancies could be selectively induced in WSe_2_ monolayers, giving rise to a greatly prolonged exciton radiative lifetime of ~200 ns and a valley polarization lifetime of ~1 µs. However, it is important to control the species of generated defects, which is rather technically challenging. While many species of defects can accelerate the relaxation process of photocarriers, certain kinds of defects may work differently and inhibit the relaxation process. A first-principle calculation on monolayer WSe_2_ revealed that Se vacancies could help suppress the phonon (mainly A_1g_ mode)-assisted recombination by breaking the lattice symmetry and reducing the intensity of the A_1g_ mode, giving rise to an increase in the recombination time constant from ~400 ps to ~3.1 ns^72^. Due to the difficulty in controlling not only the amount but also the species of point defects, it remains challenging to use particle irradiation as a practical method for tuning photocarrier dynamics.

In addition to bombarding samples to generate intrinsic defects, introducing extrinsic defects by element doping is another approach that could be adapted from the experience with bulk semiconductors. The advantage of this approach is the potential to achieve better crystallinity of the samples. As an example, for Cd_3_As_2_ films, which have been widely studied as 3D topological Dirac semimetals, a long-lived relaxation component ranging from 200 ps to 2.8 ns was recently observed in Mn-doped samples, which was attributed to the relaxation of photocarriers from the impurity band to the valence band^73^. However, the research on element doping of 2D systems and the resulting impact on photocarrier dynamics remains limited, probably hindered by the difficulty in doping atomically thin samples. In a recent study on Re-doped MoS_2_, it was shown that the exciton lifetime was reduced by ~20 times (from ~22 ps to ~1 ps) at a low doping level of 0.6%, which was attributed to the efficient Auger recombination assisted by Re dopants^74^.

On the other hand, robust approaches to suppress the effects of defect states are also essential for effective control of photocarrier dynamics. Recently, it was reported that the defect trapping time in monolayer MoS_2_ is increased from 3 to 8 ps at 0.6% strain (Fig. 5c). This modification corresponds to an increase of 440% per percent strain, whose mechanism needs to be further investigated^75^. In a recent study by Fu et al., *h*BN encapsulation was also reported to suppress the participation of defect states at the surface and interface in the photocarrier relaxation process. By exploiting this strategy, the non-radiative recombination lifetime of monolayer WS_2_ has been increased from ~24 ps for bare WS_2_ to ~48 ps for *h*BN/WS_2_ and ~86 ps to *h*BN/WS_2_/*h*BN (Fig. 5d)^42^.

### Phonon-assisted relaxation

The participation of phonons has an essential role throughout the relaxation process of photocarriers by taking a significant percentage of released energy and fulfilling the requirement of momentum conservation. For 2D materials, the role that phonons play could be more significant. On the one hand, the coupling between charge carriers and phonons can be enhanced due to the suppressed dielectric screening; on the other hand, the high surface-to-volume ratio makes 2D materials more susceptible to the external phononic environment.

**Fig. 6:**
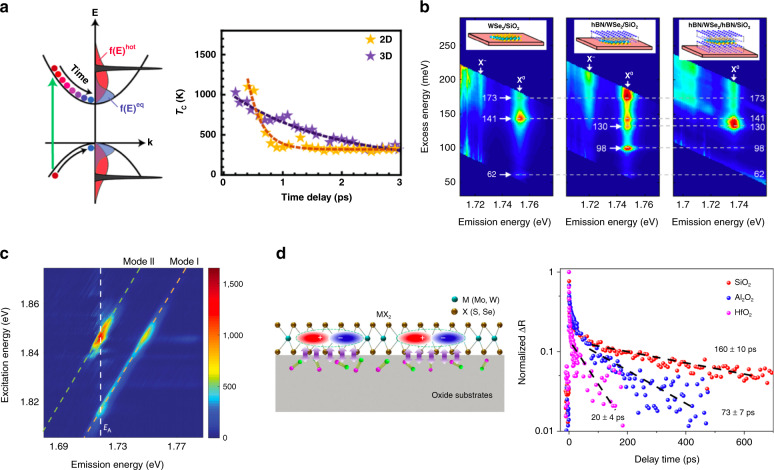
Phonon-assisted photocarrier relaxation. **a** Photoexcited charge carrier cooling in methylammonium lead iodide (MAPI) nanoplatelets (NPls). (Left) Scheme of the thermalization and relaxation of photoexcited electrons and holes in the one-particle picture. (Right) Transients of the calculated carrier temperature *T*_c_ (cooling curves) for quasi-3D and 2D samples. **b** (From left to right) PLE intensity plots of WSe_2_/SiO_2_, *h*BN/WSe_2_/SiO_2_, and *h*BN/WSe_2_/*h*BN/SiO_2_ samples at 5 K. **c** Raman excitation spectrum of the WSe_2_/*h*BN heterostructure at 77 K. **d** Ultrafast photocarrier dynamics of monolayer MoSe_2_ on different oxide substrates. (Left) Illustration of interfacial electron–phonon (*e–ph*) coupling. (Right) Photocarrier dynamics of monolayer MoSe_2_ on different substrates. Figure reproduced with permission from **a** ref. ^76^, © 2018 American Chemical Society; **b** ref. ^79^, © 2017 American Chemical Society; **c** ref. ^80^, © 2016, Springer Nature; **d** ref. ^47^, © 2019 Springer Nature

For polar semiconductors, the Fröhlich electron–LO–phonon interaction mediated by Coulomb interactions is much less screened as the thickness of the sample is reduced from the bulk to the atomic level. As a consequence, the relaxation of hot carriers to the band edges is more efficient in 2D semiconductors than in bulk semiconductors, despite the reduced number of phonons and density of states with the reduction of thickness. Such phenomena have been observed in the relaxation of photocarriers in 2D halide perovskite nanoplatelets. As shown in Fig. 6a, the time constant of hot carrier cooling in methylammonium lead iodide (MAPbI_3_) nanoplatelets was reduced from 1.7 ps to 240 fs as the thickness was reduced from ~15 to ~2 nm. In addition to the enhanced electron–phonon interaction, the more efficient heat transfer to the environment due to the high surface-to-volume ratio also contributes to the accelerated relaxation^76^. The electron–phonon coupling can also be modulated by modifying the sample lattice. In a recent study, a shortening of the photocarrier relaxation time by an order of magnitude was achieved for Cd_3_As_2_ films with 2% Cr doping. The theoretical simulation found that the tuning was due to the opening of the bandgap produced by doping-induced changes in the lattice symmetry, which activated an additional phonon scattering channel for relaxation^77^.

Moreover, owing to the atomic thickness, phonons from the surrounding environment can interact with photocarriers in the 2D samples directly. The efficient interlayer coupling between excitons in 2D semiconductors and phonons in the environment has also been revealed by various optical and electrical measurements^78–80^. It has been observed that the excitons of monolayer WSe_2_ samples interact strongly with phonons in surrounding *h*BN layers or dielectric substrates (SiO_2_, Al_2_O_3_)^79,80^. As shown in Fig. 6b^79^ and c^80^, the coupling manifests as pronounced resonance features in the PL spectra of WSe_2_ excitons or in the Raman spectra of environmental phonons when the energy difference between excitation and emission matches the energy of phonons.

Recently, by placing MoSe_2_ monolayers on three kinds of dielectric layers (SiO_2_, Al_2_O_3_, and HfO_2_), wide-range tuning of the non-radiative recombination lifetime of excitons was achieved, from 160 ± 10 ps for MoSe_2_/SiO_2_ to 20 ± 4 ps for MoSe_2_/HfO_2_ (Fig. 6d). The mechanism underlying this modulation has been identified as the interfacial electron–phonon coupling, where variations in the coupling strength and number of participating phonons lead to differences in the non-radiative recombination dynamics^47^.

In the same study by Fu et al., it was found that the dynamics of exciton formation also varied under different conditions of *h*BN encapsulation. The formation time was shortened from 1.35 to 0.69 ps from the *h*BN/WS_2_ arrangement to the *h*BN/WS_2_/hBN arrangement. Such facilitation of exciton formation has been attributed to the introduction of interfacial phonon modes by the capping *h*BN layer. Meanwhile, the *h*BN inserted under the WS_2_ layer slowed down the exciton formation (from 0.89 to 1.35 ps) by suppressing the charge transfer and doping effect from the substrate. In addition, the extracted diffusion coefficient of the excitons was reduced by approximately four times from the bare WS_2_ monolayer to the fully encapsulated monolayer due to the scattering by additional phonons introduced by *h*BN^42^. In a few studies on graphene, it has been found that the photocarrier lifetimes of CVD-grown graphene transferred to *h*BN are shorter than those of graphene transferred to SiO_2_^81^, and photocarriers in graphene epitaxially grown on SiC substrates relax faster than those in graphene transferred to SiC substrates^82,83^. These discrepancies have been attributed to the difference in the coupling between photocarriers in the graphene layers and phonons in the substrates. The shorter distances between graphene and the substrates due to smoother *h*BN and direct synthesis enable more efficient coupling, leading to faster relaxation of non-equilibrium photocarriers.

### Photocarrier recombination in vdW heterostructures

**Fig. 7:**
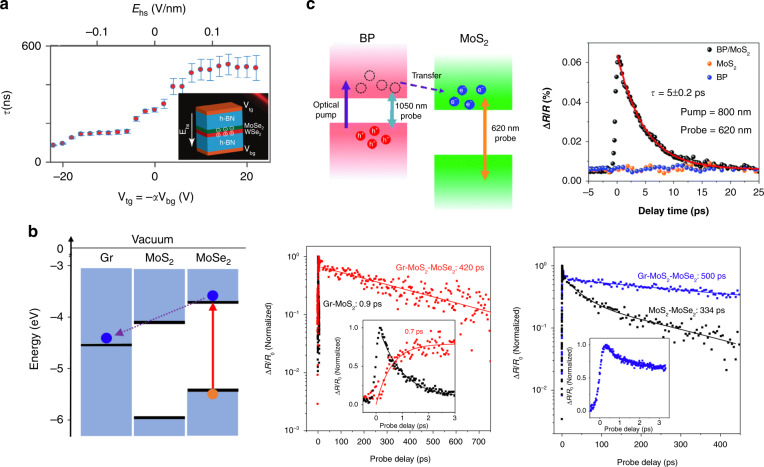
Photocarrier relaxation in vdW heterostructures. **a** Interlayer exciton (radiative) lifetime of a MoSe_2_/WSe_2_ heterostructure as a function of the electric field applied across the heterostructure. The inset shows a schematic of the heterostructure cross-section. **b** (Left) Band alignment of the graphene/MoS_2_/MoSe_2_ trilayer sample. (Middle) Electron transfer from MoSe_2_ to graphene and its lifetime in the trilayer. (Right) Hole dynamics in the trilayer. **c** (Left) Schematic illustrating the transient processes occurring in a BP/MoS_2_ heterostructure under different pump-probe conditions. (Right) Transient reflectivity signals of the BP/MoS_2_ heterostructure and MoS_2_ when pumped at 800 nm and probed at 620 nm, comparing the lifetimes of electrons. Figure reproduced with permission from **a** ref. ^84^, © 2019 AAAS; **b** ref. ^86^, © 2019 Royal Society of Chemistry; **c** ref. ^87^, © 2018 IOP Publishing

In vdW heterostructures, the electrons and holes are confined in different layers and bound as interlayer excitons after charge transfer, allowing manipulation of the separation between them. When the electron–hole separation of the excitons is altered, the changes in the overlap between electron and hole wave functions result in a change in the exciton oscillator strength, thus affecting the recombination rate. In a study on a MoSe_2_/WSe_2_ heterostructure, when an out-of-plane electric field was applied across the heterostructure antiparallel to the interlayer exciton dipole moment, the radiative recombination lifetime of the interlayer exciton was increased (Fig. 7a). As the electric field pulls the electrons and holes of the interlayer excitons apart, the probabilities that they appear at the same positions and recombine are reduced^84^.

By carefully designing the layered structure and the resulting band alignment, wide-range tuning (mostly elongation) of photocarrier lifetimes can be achieved. In a study on TMD heterostructures, WSe_2_, MoSe_2_, WS_2_, and MoS_2_ monolayers were stacked vertically one by one to form heterostructures. With this design, the photogenerated electrons and holes reside at the top and bottom TMD monolayers after charge transfer. It was shown that from the WSe_2_ monolayer to the WSe_2_/MoSe_2_/WS_2_/MoS_2_ four-layer heterostructure, both the initial charge transfer and the subsequent relaxation of transferred electrons are slowed down. The lifetime of photoexcited electrons was increased from ~100 ps to several nanoseconds^85^. In another example, this approach was employed to enhance the photocarrier lifetime in graphene, which has been challenging, limiting the application of graphene in many optoelectronic devices. Band alignment in vdW heterostructures is designed to make the photocarriers generated in TMD layers transfer to and reside in a graphene layer and recombine with the opposite type of charge carrier left in the TMD layer. In the case of *n*-type doping shown in Fig. 7b, electrons generated in the MoSe_2_ layer transfer to the graphene layer and demonstrate ultraslow recombination (~420 ps) with holes residing in MoSe_2_. In contrast, photocarriers generated in graphene recombine on an ultrashort timescale (~0.9 ps). In this way, the lifetime of carriers in graphene is enhanced by two orders of magnitude^86^.

On the other hand, when electrons and holes are less tightly bound, the motion of carriers in real space can also result in the modification of their relaxation dynamics. In a recent study employing black phosphorus (BP), which has a small exciton binding energy (<40 meV), to form a type-II heterostructure with MoS_2_, separated photoexcited electrons and holes existed as (quasi)free charge carriers across the vdW interface. As shown in Fig. 7c, these separated photocarriers demonstrate an unusually short lifetime (~5–6 ps), much shorter than those for individual BP or MoS_2_ layers. It has been demonstrated that the recombination dynamics can be well described by a Langevin recombination model, which is mediated by the Coulomb interactions between electrons and holes across the interface. The higher mobility and larger density of photocarriers in BP increase the chance that the electrons and holes meet within the Coulomb capture radius and recombine with each other^87^.

### Transition among different quasiparticles

In addition to neutral excitons, in real samples and devices, it is inevitable that other types of quasiparticles, such as trions and biexcitons, as well as free carriers, coexist and undergo transitions among each other, which further complicates the relaxation dynamics of non-equilibrium photocarriers. For example, combining with free carriers to form trions represents an important non-radiative decay channel of neutral excitons, which reduces the radiative lifetime of neutral excitons and limits the QY^88^. Therefore, when the ratios between different quasiparticles in samples are altered by means of doping of free carriers, the relative portions of different relaxation pathways, and thus the transient optical responses of the whole sample, can be modified.

**Fig. 8:**
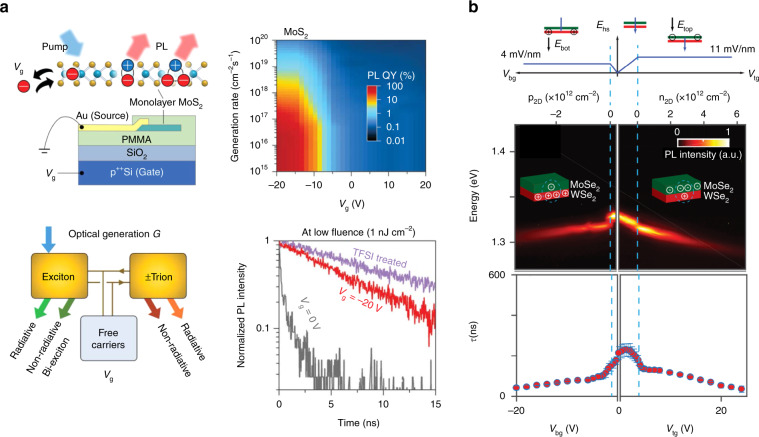
Transitions among different quasiparticles. **a** Near-unity photoluminescence (PL) quantum yield in MoS_2_ through electrostatic doping compensation. (Top left) Schematic showing control of different quasiparticles by back-gate voltage *V*_g_ and photocarrier generation rate *G*. (Top right) MoS_2_ PL QY versus *G* and *V*_g_. (Bottom left) Proposed exciton and trion recombination pathways in TMD materials. (Bottom right) Time-resolved PL of TFSI-treated MoS_2_ (purple) and a MoS_2_ device under *V*_g_ = 0 V (black) and *V*_g_ = –20 V (red) at a pump fluence of 1 nJ/cm^2^. **b** Carrier density control of interlayer excitons in a MoSe_2_/WSe_2_ heterostructure. (Top) The calculated electric field across the heterostructure *E*_hs_ versus the top and bottom gate voltages (*V*_tg_ and *V*_bg_). The top cartoons represent the heterostructure for different applied gate voltages. (Middle) Single-gate dependence (*V*_tg_ or *V*_bg_) of the PL showing the formation of charged interlayer excitons with varying carrier density obtained from the gate operation scheme in the top panel. (Bottom) Neutral and charged interlayer exciton lifetime *τ* versus carrier density. Figure reproduced with permission from **a** ref. ^89^., © 2019 AAAS; **b** ref. ^84^, © 2019 AAAS

Tuning the background free carrier density is an effective approach to modulate these transitions, which can be done by electrostatic^89,90^ or chemical doping^9^ or the combination of both to achieve a broader controllable regime^91^. In the work by Lien et al., effective suppression of non-radiative recombination in MoS_2_ was achieved using an electrostatic doping strategy. As shown in Fig. 8a, by tuning the electrical bias, a near-unity QY and a two-order-of-magnitude increase in the exciton radiative lifetime were achieved. A phenomenological model was developed to interpret the tuning mechanism, in which dynamic interactions between excitons, trions, and free carriers, and their respective recombination processes, including both radiative and non-radiative ones, were taken into account. By fitting the measured data to this model, it was found that at a relatively low photogeneration rate, the non-radiative recombination of trions was much more efficient than the radiative recombination of both neutral excitons and trions^89^. In the chemical doping case, exfoliated monolayer MoS_2_ samples were immersed in a non-oxidizing organic superacid, a bis (trifluoromethane) sulfonimide (TFSI) solution, and a hundred-fold enhancement in the PL intensity was achieved, which was attributed to hole doping through surface charge transfer^9^. The radiative lifetime of excitons has been increased by over two orders of magnitude by TFSI treatment, even larger than what has been achieved by a negative gate bias of −20 V^89^.

In a work on a MoSe_2_/WSe_2_ heterostructure, by controlling the gate voltage applied to each TMD layer, the electrostatic doping conditions in these two layers could be tuned independently. As shown in Fig. 8b, emission of interlayer trions is observed when the doping concentration in either layer is above a certain threshold, and the radiative lifetime is decreased with increasing doping concentration in each layer^84^.

In addition to radiative recombination, it has recently been found that the non-radiative recombination dynamics of neutral and charged excitons can also exhibit discrepant features, as shown in a back-gated WS_2_ monolayer^92^.

## Valley/spin polarization dynamics of photocarriers in 2D TMDs

To be exploited as an alternative to charges for information storage and processing, the valley/spin polarization of carriers in monolayer TMDs needs to have a sufficiently long lifetime. However, it has been demonstrated both experimentally^93,94^ and theoretically^95,96^ that for neutral excitons in monolayer TMDs, it is rather challenging to maintain a long lifetime of valley/spin polarization due to the strong electron–hole exchange interaction. Hence, efforts to prolong the lifetime of carriers’ valley/spin polarization mainly involve reducing the Coulomb electron–hole exchange interaction and, more importantly, exploring carrier species with non-zero momentum/spin.

**Fig. 9:**
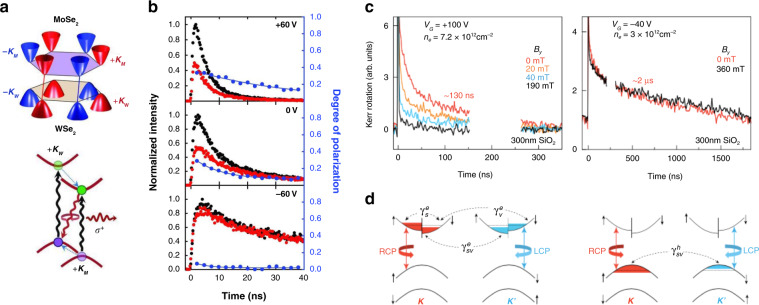
Valley/spin polarization dynamics of photocarriers in 2D TMDs. **a** (Top) Illustration of the Dirac points in the hexagonal Brillouin zone of a MoSe_2_/WSe_2_ heterostructure. (Bottom) Schematic of the interlayer exciton in the +K valley of the heterostructure. **b** Time-resolved interlayer exciton PL at selected gate voltages. **c** (Left) Time-resolved Kerr rotation (TRKR) at 5 K on gated monolayer WSe_2_ (left) in the heavily electron-doped regime at the negatively charged exciton transition and (right) in the hole-doped regime at the positively charged exciton transition. **d** Diagrams depicting the simplest WSe_2_ band structure in the (left) electron-doped and (right) hole-doped regimes, along with available scattering pathways. Figure reproduced with permission from **a**, **b** ref. ^98^, © 2016 AAAS; **c**, **d** ref. ^101^, © 2017 American Physical Society

Due to valley/spin conservation during the ultrafast interlayer charge transfer process^97^, interlayer excitons in vdW heterostructures are also able to store valley/spin polarization. Rivera et al. investigated the valley polarization dynamics of bright interlayer excitons in a MoSe_2_/WSe_2_ heterostructure by circular polarization-resolved PL. As illustrated in Fig. 9a, with close lattice constants and a small interlayer twist angle between the two TMD layers, the valleys in their Brillouin zone are nearly aligned. Excitation by *σ*^+^ light generates photocarriers in the +K valleys of the MoSe_2_ and WSe_2_ layers, which can form interlayer excitons in the +K valleys after ultrafast charge transfer and can recombine to emit photons with *σ*^+^ helicity. The degree of polarization demonstrates a lifetime as long as ~40 ns under a gate voltage of +60 V (Fig. 9b), which has been attributed to the suppression of the Coulomb exchange interaction and interlayer recombination induced by the increased separation in both real and momentum spaces^98^.

On the other hand, the depolarization of charge carriers with non-zero total momentum/spin, such as (both intralayer and interlayer) trions^99,100^, resident carriers^101–103^, and dark excitons^104,105^, involves intervalley scattering or spin-flip and thus demonstrates a lower rate. In the study by Yan et al. on monolayer WSe_2_, the valley polarization dynamics of neutral excitons, trions, and free carriers were distinguished by varying the probing energy of the time-resolved Kerr rotation. The trions and free carriers exhibit valley polarization lifetimes of ~130 ps and ~2 ns at 70 K, much longer than that of the neutral excitons (~2 ps) owing to the suppressed intervalley scattering^103^.

Knowing the advantages of charged quasiparticles, electrostatic doping can be employed to further tune the valley polarization dynamics. In another study on WSe_2_ monolayers, time-resolved Kerr rotation measurements demonstrated an ~130 ns lifetime for negatively charged trions and an ~2 µs lifetime for positively charged trions under *n*- and *p*-doped regimes, respectively (Fig. 9c). In contrast, the valley-polarized excitons and trions scatter and recombine within 30 ps under zero gate bias. This long-lived valley polarization has been attributed to the resident carriers after trion recombination: the excited trions recombine on a short timescale and transfer the non-zero valley/spin polarization to the remaining electrons or holes. As shown in Fig. 9d, for the case of resident electrons, their valley/spin polarization can be relaxed through spin-conserving intervalley scattering ($\gamma _{\mathrm{v}}^e$), spin relaxation within a valley ($\gamma _{\mathrm{s}}^e$), and spin-flip intervalley scattering ($\gamma _{\mathrm{sv}}^e$). Those processes involving valley or spin scattering would lead to longer valley/spin polarization lifetimes. Moreover, for resident holes, the first two processes are effectively suppressed by the giant spin-orbit splitting of the valence band; thus, spin and valley scattering must occur simultaneously for the polarized holes to relax, giving rise to an even longer lifetime, which is also less sensitive to an applied magnetic field^101^.

In TMD-based heterostructures, the valley/spin dynamics of electrons or holes in different layers can be probed separately, and the majority carrier exhibits longer lifetimes of both the population and valley/spin polarization. In the study by Kim et al., for a WSe_2_/MoS_2_ heterostructure, it was shown that the population and polarization of holes are not limited by those of electrons, demonstrating a population lifetime of ~1 µs and a valley depolarization lifetime of ~40 µs at 10 K^106^. A further study from the same group reported tuning by electrostatic doping. In the neutral and *n*-doped regimes, the valley polarization lifetime of holes is limited by the population lifetime of the total excess holes. In contrast, the hole valley polarization decouples from the total excess hole population in the *p*-doped regime and exhibits a longer lifetime. According to the explanation given in that study, for the electron-doped heterostructures, the majority of electrons recombine with the valley-polarized holes; thus, the valley polarization lifetime of holes is determined by the recombination dynamics. In the hole-doped case, the original hole density is larger than that of the photogenerated holes, and the minority electrons recombine with holes in both the +K and −K valleys equally; thus, the hole polarization is not changed by the interlayer recombination^107^.

## Conclusion and perspectives

In this review, we have summarized the progress in understanding and manipulating the photocarrier relaxation dynamics in 2D semiconductors. It can be seen that while the reduced thickness of 2D systems impairs the effectiveness of modulation methods developed for bulk semiconductors, it also provides new physical knobs that can be tuned and even new degrees of freedom that can be utilized in devices. The aforementioned achievements have made 2D semiconductors more relevant for real applications.

Moreover, advances in synthesis and processing techniques, as well as the discovery of exotic phenomena in 2D semiconductors, continue to suggest new strategies to this end. Recently, by using chemical vapor transport (CVT) and the self-flux growth method, single crystals of MoSe_2_ and WSe_2_ were prepared with the density of intrinsic point defects being reduced from ~10^13^/cm^2^ to as low as 10^11^/cm^2^ ^108^. The capacity to reduce the defect density or passivate the defect states not only provides high-quality samples with suppressed non-radiative recombination and high QY but also makes high-density doping through electrostatic or optical methods possible^58^. More precise control of the sample composition over a wider range while maintaining the crystallinity will enable convenient property tailoring, as in the case of bulk semiconductors. For TMDs, it has been shown experimentally and theoretically that more complicated compound systems, such as trinary compounds^109^ and Janus TMD systems (MXY, X, Y = S, Se, and Te; X ≠ Y)^110^, are of great potential for realizing significantly modified ultrafast relaxation dynamics. It has been predicted that the Janus-MoSTe would exhibit an ultralong lifetime (~1.3 ns) due to the large spatial separation and reduced binding energy^111^. These advances in sample preparation techniques, while still limited, hold great promise for leading to approaches that can meet the requirements of real optoelectronic and photonic devices. In recent years, the moiré pattern, which is formed in vdW heterostructures with small lattice mismatches and twist angles, has been shown to modify the physical properties of interlayer excitons in TMD-based heterostructures over a long translation period^5^. Modulation of properties such as the diffusivity and spin/valley polarization through the moiré potential has been demonstrated^59,112^. However, studies on modulating the photocarrier relaxation in 2D semiconductors are still at a relatively early stage, and robust approaches to realize reliable and wide-range tuning of the photocarrier relaxation behavior, including both relaxation pathways and temporal dynamics, remain limited. Tremendous research efforts are still needed in both improvement of the fundamental understanding and practical modulation of the photocarrier relaxation in 2D semiconductors.
